# Supplementary material for: Transgenerational and Molecular Responses to Lanthanum Exposure in a Spodoptera littoralis-Brassica rapa System
Source: Int J Mol Sci. 2025 Aug 30;26(17):8462. doi: 10.3390/ijms26178462 (PMC12429623; doi:10.3390/ijms26178462)
Supplement: Supplementary file 1 [file ijms-26-08462-s001.zip › ijms-3828501-supplementary.pdf]

Supplementary Table S1. Expression of Selected Genes in Brassica.rapa Exposed to Lanthanum for 7 Days and Subsequently Infested with Spodoptera. littoralis for 24 Hours. Log_2(Fold Change) Indicates Relative Gene Expression Compared to the Control (No S¡.littoralis.Infestation and No Lanthanum Treatment). ns: p > 0.05; .: 0.05 < p < 0,1; *: p < 0.05, **: p < 0.01, ***: p < 0.001; Reference gene is GADPH

Categories	Gene ID	Gene Code	Gene description	La_0µM	La_1µM	La_1mM	La_10mM
Calcium Signaling/Sensing	LOC103831104	CaM1	Calmodulin 1	-0.12 ± 0.01	-0.16 ± 0.01	**-0.37 ± 0.03***	-0.07 ± 0.01
Calcium Signaling/Sensing	LOC103847998	CML30	Calmodulin-like protein 30	0.02 ± 0.00	**-0.31 ± 0.01***	-0.14 ± 0.06	**-0.67 ± 0.06***
Calcium Signaling/Sensing	LOC103861199	CML42	Calmodulin-like protein 42	**0.07 ± 0.00***	0.18 ± 0.02	**-0.11 ± 0.00****	-0.09 ± 0.00
Calcium Signaling/Sensing	LOC103848449	CML43	Calmodulin-like protein 43	**-0.57 ± 0.00****	**-0.35 ± 0.01****	**-0.69 ± 0.02*****	**-0.51 ± 0.01*****
Calcium Signaling/Sensing	Bra002472	CAMK	CaM-dependent protein kinases	-0.17 ± 0.02	-0.10 ± 0.01	-0.28 ± 0.04	-0.09 ± 0.01
Calcium Signaling/Sensing	LOC103857828	CDPK20	calcium-dependent protein kinase 20	**0.15 ± 0.00****	**0.18 ± 0.01***	**0.16 ± 0.01***	**0.18 ± 0.00****
Calcium Signaling/Sensing	LOC103851852	CBL2	calcineurin B-like protein 2	**0.92 ± 0.16****	**0.99 ± 0.15****	**0.82 ± 0.16***	-0.03 ± 0.00
Calcium Signaling/Sensing	LOC103869680	CIPK1	CBL interacting protein kinase 1	0.0042 ± 0.00	**0.33 ± 0.02****	0.11 ± 0.01	0.16 ± 0.01
Oxidative Stress	LOC106387767	SOD	Superoxide dismutase [Cu-Zn] SOD	0.17 ± 0.05	0.04 ± 0.01	0.17 ± 0.02	0.04 ± 0.01
Oxidative Stress	LOC103831878	CAT2	Catalase-2	0.34 ± 0.04	**0.52 ± 0.05***	**0.60 ± 0.07***	0.26 ± 0.04
Oxidative Stress	LOC103835788	CAT3	Catalase-3	0.02 ± 0.00	-0.24 ± 0.03	-0.02 ± 0.00	-0.04 ± 0.00
Oxidative Stress	LOC103871533	APX1	L-ascorbate peroxidase 1	**0.71 ± 0.19****	**1.41 ± 0.39****	**-0.18 ± 0.06***	**-0.17 ± 0.05***
Oxidative Stress	LOC106312869	POX32	Peroxidase 32	0.10 ± 0.01	0.10 ± 0.00	0.06 ± 0.00	**0.23 ± 0.01***

Categories	Gene ID	Gene Code	Gene description	La_0µM	La_1µM	La_1mM	La_10mM
Oxidative Stress	LOC103828679	GR1	Glutathione reductase 1	0.72 ± 0.19	**-0.38 ± 0.01****	**-0.43 ± 0.01****	**-0.52 ± 0.03****
Oxidative Stress	LOC103852942	DHAR2	glutathione S-transferase 2	**-0.21 ± 0.01***	-0.08 ± 0.01	-0.04 ± 0.00	0.02 ± 0.00
Oxidative Stress	LOC103853516	GPX6	glutathione peroxidase 6	-3.87 ± 2.08	-2.66 ± 1.99	-3.18 ± 0.67	-2.28 ± 1.09

Supplementary Table S2. The list of primers (source* https://www.ncbi.nlm.nih.gov/)

Categories	Gene Names	Gene Locus* (NCBI)	Functions/Description	Forward	Reverse
Calcium Signaling/Sensing (*Brassica rapa*)	CaM1	LOC103831104	Calmodulin 1	GATGAAGAAGTTGAGGAGA	TCACTTAGCCATCATAATCT
Calcium Signaling/Sensing (*Brassica rapa*)	CML30	LOC103847998	Calmodulin-like protein 30	AGAGAGAGCGATCCAAGACG	CCGACATCACTTCTTCTGCG
Calcium Signaling/Sensing (*Brassica rapa*)	CML42	LOC103861199	Calmodulin-like protein 42	TCTCCGACCTCAAATCCACC	TCAAAACAGCCTGCAACTCC
Calcium Signaling/Sensing (*Brassica rapa*)	CML43	LOC103848449	Calmodulin-like protein 43	CATCTGCGAGCTCAAACCAA	ACCTTCTGAAGTTCCTCGGG
Calcium Signaling/Sensing (*Brassica rapa*)	CAMK	Bra002472	CaM-dependent protein kinases	ACGTATAGTGGGAGAGCTGC	ATCCGGGATGTTCAAAGGGT
Calcium Signaling/Sensing (*Brassica rapa*)	CDPK20	LOC103857828	calcium-dependent protein kinase 20	CGCTGTAGGTGTTCATGTCG	CGCAGCTTCTTCATCTCCAC
Calcium Signaling/Sensing (*Brassica rapa*)	CBL2	LOC103851852	calcineurin B-like protein 2	CTCGTGCTCTCTCCGTCTTC	AGATTCAGCAAGCGTAGCCA
Calcium Signaling/Sensing (*Brassica rapa*)	CIPK1	LOC103869680	CBL interacting protein kinase 1	AAGCAAGAAGAACATAACCAGAAGG	CTAGCTCTTGTTCCGGCGAG
Oxidative Stress (*Brassica rapa*)	SOD	LOC106387767	Superoxide dismutase [Cu-Zn] SOD	TCACACAAGACACTTCCGGT	ACCAGTAGAGTTGCAGCCAT
Oxidative Stress (*Brassica rapa*)	CAT2	LOC103831878	Catalase-2	GCTTGCCTTCTGTCCTGCTA	CGGTGTCTCTGAGTATCGGC

Oxidative Stress (*Brassica rapa*)	CAT3	LOC103835788	Catalase-3	TCTCCAGCTGCCTGTGAATG	TAGGAACTTTCTCGGCGCAG
Oxidative Stress (*Brassica rapa*)	APX1	LOC103871533	L-ascorbate peroxidase 1	GTTGTGACCACTTGAGGCAG	CCAGACCTATCCTTGTGGCA
Oxidative Stress (*Brassica rapa*)	POX32	LOC106312869	Peroxidase 32	CCTCGAGTCAGATGGTGTGT	CGCAACCCTTTTACGAACCA
Oxidative Stress (*Brassica rapa*)	GR1	LOC103828679	Glutathione reductase 1	TCTCGTCATCAAAGCCCCTT	ACCGCCAAGCACATTTTGAT
Oxidative Stress	DHAR2	LOC103852942	glutathione S-transferase 2	CAGCGACCACAAACTCCTTC	TCACTGCGGTTGATCTGAGT
Oxidative Stress (*Brassica rapa*)	GPX6	LOC103853516	glutathione peroxidase 6	CTCTCGACTCCCATCTAAGTTC	GCTTAGATCAACATCGTTTCCC
House keeper gene (*Brassica rapa*)	GAPHD	LOC103848938	Glyceraldehyde-3-phosphate dehydrogenase	TTCCCACCGTTGATGTCTCA	CACGACACAAGCTTCACGAA
Oxidative stress-related gene (*Spodoptera sp.*)	SOD	LOC118271250	Superoxide dismutase-like	GCAGCAGTGTTTGGACACAT	GCACAAACATCAGTCCAGGT
Oxidative stress-related gene (*Spodoptera sp.*)	CAT	LOC111360747	Catalase	TCTCCACTGTTGGTGGTGAA	GTTGCCAACAAGATCCCAGT
Oxidative stress-related gene (*Spodoptera sp.*)	GST1	LOC111364724	Glutathione S-transferase 1-like	CAGTAAATTGACGGCCTGGT	CTTCAGTTTGGACTGCACGA
House keeper gene (*Spodoptera sp.*)	ACT	LOC111364589	Spodoptera litura actin	GATCTGGCACCACACCTTCT	TCTTCTCCCTGTTGGCCTTA

SUPPLEMENTARY DATA SET S1

	Larvae gain weight (%)		
tmt	48hr	72hr	7days
Control	176.0	243.1	693.6
Control	360.7	364.2	712.8
Control	126.4	218.7	817.3
Control	222.8	361.6	822.6
La_1 μM	74.6	118.3	586.6
La_1 μM	53.3	92.6	832.3
La_1 μM	72.7	227.1	998.2
La_1 μM	117.5	228.2	772.0
La_1 mM	98.5	223.1	887.9
La_1 mM	78.4	189.9	871.4
La_1 mM	75.9	144.2	683.9
La_1 mM	93.3	112.7	627.2
La_10 mM	111.3	190.5	553.9
La_10 mM	103.9	142.8	632.7
La_10 mM	135.2	145.3	513.9
La_10 mM	108.1	160.6	528.6

	Larvae survival (%)		
tmt	48hr_sur	72hr_sur	7days_sur
Control	66.7	66.7	66.67
Control	100	100	66.67
Control	100	100	100
Control	100	100	100
La_1 μM	66.7	100	66.67
La_1 μM	100	66.7	66.67
La_1 μM	100	66.7	66.67
La_1 μM	100	100	100
La_1 mM	100	66.7	66.67
La_1 mM	100	100	100
La_1 mM	66.7	100	66.67
La_1 mM	66.7	66.7	66.67
La_10 mM	66.7	66.7	66.67
La_10 mM	100	66.7	66.67
La_10 mM	66.7	66.7	33.33
La_10 mM	66.7	66.7	66.67

tmt	[illegible] (mg/gram)	H2O2 (uM/mg)
Control	11.868	1.14
Control	11.821	0.69
Control	10.837	0.58
Control	12.946	0.54
La_1 μM	10.749	0.51
La_1 μM	11.759	0.69
La_1 μM	10.384	1.10
La_1 μM	10.103	0.51
La_1 mM	12.634	0.54
La_1 mM	11.759	0.73
La_1 mM	12.618	0.81
La_1 mM	12.321	1.07
La_10 mM	12.852	0.88
La_10 mM	15.226	0.58
La_10 mM	14.211	0.66
La_10 mM	14.555	0.66

Tmt	Pupae rate	[illegible] weight (mg)	[illegible] male pupae (%)
Control	50	0.31	50.0
Control	25	0.35	100.0
Control	25	0.28	100.0
Control	25	0.37	100.0
Control	50	0.31	100.0
Control	25	0.33	100.0
Control	25	0.29	100.0
Control	50	0.33	50.0
Control	25	0.25	100.0
La_10 mM	50	0.35	50.0
La_10 mM	75	0.37	66.7
La_10 mM	75	0.28	100.0
La_10 mM	50	0.33	50.0
La_10 mM	100	0.29	50.0
La_10 mM	50	0.31	50.0
La_10 mM	50	0.41	50.0
La_10 mM	50	0.32	50.0

Tmt	Weight gain [%]	Survival [%]
	48hrs	48hrs
CT_CT4	125.55	100
CT_CT5	141.84	75
CT_CT6	116.66	75
CT_CT7	94.42	75
CT_CT8	77.79	100
CT_CT9	70.05	100
CT_La1	77.17	75
CT_La2	70.88	75
CT_La3	69.64	75
CT_La4	59.86	75
CT_La5	59.02	75
CT_La6	83.99	75
CT_La7	64.74	75
CT_La8	80.71	
La_La1	81.79	75
La_La2	131.75	100
La_La3	124.54	75
La_La4	90.83	100
La_La5	82.21	100
La_La6	73.30	75
La_La7	91.53	75
La_La8	87.16	75
La_La9	78.98	100
La_CT1	96.74	100
La_CT2	64.82	75
La_CT3	60.85	75
La_CT4	134.06	75
La_CT5	115.04	100
La_CT6	119.15	100
La_CT7	70.23	100

tmt	weighgain (%)	H2O2	Protein
AD_La0	82.00	0.40	13.9925
AD_La0	52.73	0.36	13.27
AD_La0	66.27	0.62	14.57045
AD_La0	71.83	0.21	15.33583
AD_La0	87.10		
La_0	81.22	1.14	10.55608
La_0	61.89	0.66	10.806
La_0	57.83	0.88	10.77476
La_0	82.62	1.10	11.80569
La_0	65.13		
La_1 μM	76.33	1.70	12.07123
La_1 μM	65.85	1.40	12.86265
La_1 μM	97.17	1.18	13.30522
La_1 μM	101.67	1.55	13.2115
La_1 μM	113.97		
La_1 mM	53.35	1.51	12.02437
La_1 mM	59.65	1.25	12.71165
La_1 mM	44.14	1.14	11.83172
La_1 mM	65.17	1.14	10.75914
La_1 mM	43.57		
La_10 mM	57.10	0.88	10.30615
La_10 mM	66.60	1.03	11.05592
La_10 mM	71.72	0.84	11.37353
La_10 mM	39.53		12.75851
La_10 mM	45.58		

tmt	Leave Biomass (gram)	Leaveprotein (mg/gram)	Chla+b (mg/gram)
Control	6.6	4.379881	8.13296
Control	10.78	4.325211	8.51512
Control	8.34	4.352546	7.75732
Control	5.61		
Control	5.91		
La_0	6.24	6.152765	8.747613
La_0	5.14	4.106529	8.42872
La_0	6.49	5.306675	9.02944
La_0	5.73	5.660731	8.78468
La_0	4.38		
La_1 μM	7.2	3.856607	9.35268
La_1 μM	5.92	3.848797	9.8854
La_1 μM	6.3	4.012808	10.00184
La_1 μM	5.07	3.427054	9.64064
La_1 μM	4.77		
La_1 μM			
La_1 mM	3.59	4.645423	9.66416
La_1 mM	4.4	4.536082	8.82008
La_1 mM	5.59	4.910965	8.60932
La_1 mM	4.47	4.465792	8.31228
La_1 mM	4.74		
La_1 mM	3.99		
La_10 mM	3.84	5.324898	9.80424
La_10 mM	3.43	5.449859	9.784173
La_10 mM	3.26	4.887535	9.74404
La_10 mM	6.54	6.137145	9.80424
La_10 mM	6.69		

Well	Well Position	Sample Name	Target Name	CT	Ct Mean
1	A1	CRTL	GADPH	22.61105	22.67423
2	A2	CRTL	GADPH	22.67541	22.67423
3	A3	CRTL	GADPH	22.73622	22.67423
61	F1	CRTL	10.CaM	26.3063	26.14098
62	F2	CRTL	10.CaM		26.14098
63	F3	CRTL	10.CaM	25.97565	26.14098
28	C4	CRTL	11.CML30	26.10476	26.28738
29	C5	CRTL	11.CML30		26.28738
30	C6	CRTL	11.CML30	26.47	26.28738
7	A7	CRTL	12.CML42	26.58199	26.53462
8	A8	CRTL	12.CML42	26.54911	26.53462
9	A9	CRTL	12.CML42	26.47276	26.53462
67	F7	CRTL	13.CML43		29.41304
68	F8	CRTL	13.CML43	29.4146	29.41304
69	F9	CRTL	13.CML43	29.41147	29.41304
34	C10	CRTL	14.CAMK	27.27323	27.25171
35	C11	CRTL	14.CAMK	26.84506	27.25171
36	C12	CRTL	14.CAMK	27.63684	27.25171
13	B1	La_0	GADPH	22.98045	23.04334
14	B2	La_0	GADPH	23.06473	23.04334
15	B3	La_0	GADPH	23.08485	23.04334
73	G1	La_0	10.CaM	26.13532	26.24021
74	G2	La_0	10.CaM	26.43835	26.24021
75	G3	La_0	10.CaM	26.14695	26.24021
40	D4	La_0	11.CML30	27.1938	26.71935
41	D5	La_0	11.CML30	26.48772	26.71935
42	D6	La_0	11.CML30	26.47651	26.71935
19	B7	La_0	12.CML42	27.00738	27.10218
20	B8	La_0	12.CML42	27.23518	27.10218
21	B9	La_0	12.CML42	27.06398	27.10218
79	G7	La_0	13.CML43	27.59715	27.59015
80	G8	La_0	13.CML43	27.63046	27.59015
81	G9	La_0	13.CML43	27.54285	27.59015
46	D10	La_0	14.CAMK	27.62957	27.10854
47	D11	La_0	14.CAMK	27.07712	27.10854
48	D12	La_0	14.CAMK	26.61893	27.10854
79	A1	CRTL	GADPH	23.01838	23.23205
80	A2	CRTL	GADPH	22.9806	23.23205
81	A3	CRTL	GADPH	23.69717	23.23205
4	F1	CRTL	15.BraCPK	28.85992	28.73158
5	F2	CRTL	15.BraCPK	28.52922	28.73158
6	F3	CRTL	15.BraCPK	28.8056	28.73158
19	C4	CRTL	16.BraCBL	28.763	29.20934
20	C5	CRTL	16.BraCBL	30.36666	29.20934
21	C6	CRTL	16.BraCBL	28.49838	29.20934
34	A7	CRTL	17.BraCIPK	26.68926	26.86992
35	A8	CRTL	17.BraCIPK	27.00274	26.86992
36	A9	CRTL	17.BraCIPK	26.91776	26.86992
76	B1	La_0	GADPH	23.26799	23.25186
77	B2	La_0	GADPH	23.19715	23.25186
78	B3	La_0	GADPH	23.29044	23.25186
1	G1	La_0	15.BraCPK	29.30194	29.34382
2	G2	La_0	15.BraCPK	29.23532	29.34382
3	G3	La_0	15.BraCPK	29.49421	29.34382
16	D4	La_0	16.BraCBL	36.43429	34.56501
17	D5	La_0	16.BraCBL	34.90437	34.56501
18	D6	La_0	16.BraCBL	32.35638	34.56501
31	B7	La_0	17.BraCIPK	27.17871	26.90038
32	B8	La_0	17.BraCIPK	27.23795	26.90038
33	B9	La_0	17.BraCIPK	26.2845	26.90038
1	A1	CRTL	GAPDH	24.93538	24.0859
2	A2	CRTL	GAPDH	23.5538	24.0859
3	A3	CRTL	GAPDH	23.7685	24.0859
28	C4	CRTL	2.SOD	29.48171	28.6183
29	C5	CRTL	2.SOD	28.54145	28.6183
30	C6	CRTL	2.SOD	27.83173	28.6183
7	A7	CRTL	3.CAT2	28.18135	27.75904
8	A8	CRTL	3.CAT2	27.54787	27.75904
9	A9	CRTL	3.CAT2	27.54791	27.75904
67	F7	CRTL	4.CAT3	35.47228	33.79243
68	F8	CRTL	4.CAT3	33.2764	33.79243
69	F9	CRTL	4.CAT3	32.62862	33.79243
34	C10	CRTL	5.APX	24.44855	24.34891
35	C11	CRTL	5.APX	24.48023	24.34891
36	C12	CRTL	5.APX	24.11796	24.34891
13	B1	La_0	GAPDH	24.13456	24.01144
14	B2	La_0	GAPDH	23.85231	24.01144
15	B3	La_0	GAPDH	24.04745	24.01144
40	D4	La_0	2.SOD	30.77736	29.14204
41	D5	La_0	2.SOD	28.84551	29.14204
42	D6	La_0	2.SOD	27.80327	29.14204
19	B7	La_0	3.CAT2	29.10703	28.65868
20	B8	La_0	3.CAT2	28.91133	28.65868
21	B9	La_0	3.CAT2	27.95768	28.65868
79	G7	La_0	4.CAT3	34.50225	32.9966
80	G8	La_0	4.CAT3	34.03218	32.9966
81	G9	La_0	4.CAT3	30.45536	32.9966
46	D10	La_0	5.APX	23.5119	23.58227
47	D11	La_0	5.APX	23.6908	23.58227
48	D12	La_0	5.APX	23.54412	23.58227
1	A1	CRTL	GAPHD	23.95339	23.75057
2	A2	CRTL	GAPHD	23.71069	23.75057
3	A3	CRTL	GAPHD	23.58762	23.75057
61	F1	CRTL	6.BraPOX3	28.65071	29.11954
62	F2	CRTL	6.BraPOX3	29.30635	29.11954
63	F3	CRTL	6.BraPOX3	29.40156	29.11954
28	C4	CRTL	7.BraGR1	26.52103	26.46404
29	C5	CRTL	7.BraGR1		26.46404
30	C6	CRTL	7.BraGR1	26.40704	26.46404
67	F7	CRTL	9.BraGPX6	26.69758	25.33411
68	F8	CRTL	9.BraGPX6	25.14796	25.33411
69	F9	CRTL	9.BraGPX6	24.1568	25.33411
13	B1	La_0	GAPHD	24.05272	23.87762
14	B2	La_0	GAPHD	23.60994	23.87762
15	B3	La_0	GAPHD	23.97021	23.87762
73	G1	La_0	6.BraPOX3	29.62046	29.64022
74	G2	La_0	6.BraPOX3	29.6786	29.64022
75	G3	La_0	6.BraPOX3	29.62161	29.64022
40	D4	La_0	7.BraGR1	30.65056	28.35818
41	D5	La_0	7.BraGR1	28.12962	28.35818
42	D6	La_0	7.BraGR1	26.29437	28.35818
79	G7	La_0	9.BraGPX6	24.01654	23.98575
80	G8	La_0	9.BraGPX6	23.98115	23.98575
81	G9	La_0	9.BraGPX6	23.95954	23.98575
1	A1	CRTL	GAPDH	23.88662	23.97171
2	A2	CRTL	GAPDH	23.84686	23.97171
3	A3	CRTL	GAPDH	24.18165	23.97171
61	F1	CRTL	8.BraDHAR	29.93391	30.1112
62	F2	CRTL	8.BraDHAR	30.68408	30.1112
63	F3	CRTL	8.BraDHAR	29.7156	30.1112
13	B1	La_0	GAPDH	24.24052	24.64993
14	B2	La_0	GAPDH	25.66215	24.64993
15	B3	La_0	GAPDH	24.04713	24.64993
73	G1	La_0	8.BraDHAR	30.01235	29.95988
74	G2	La_0	8.BraDHAR	29.97796	29.95988
75	G3	La_0	8.BraDHAR	29.88932	29.95988

Well	Well Position	Sample Name	Target Name	CT	Ct Mean
61	F1	AD	2_Spo_ACT	21.94223	21.9439
62	F2	AD	2_Spo_ACT	22.07271	21.9439
63	F3	AD	2_Spo_ACT	21.81676	21.9439
28	C4	AD	3_Spo_SOD	34.98409	35.27656
29	C5	AD	3_Spo_SOD		35.27656
30	C6	AD	3_Spo_SOD	35.56902	35.27656
7	A7	AD	4_Spo_CAT	22.15207	22.18227
8	A8	AD	4_Spo_CAT	22.15627	22.18227
9	A9	AD	4_Spo_CAT	22.15847	22.18227
67	F7	AD	5_Spo_GST	20.65835	20.51017
68	F8	AD	5_Spo_GST	20.73503	20.51017
69	F9	AD	5_Spo_GST	20.13713	20.51017
73	G1	CTRL	2_Spo_ACT	19.33216	19.47858
74	G2	CTRL	2_Spo_ACT	19.51668	19.47858
75	G3	CTRL	2_Spo_ACT	19.58691	19.47858
40	D4	CTRL	3_Spo_SOD	35.91652	35.44841
41	D5	CTRL	3_Spo_SOD	35.4679	35.44841
42	D6	CTRL	3_Spo_SOD	34.9608	35.44841
19	B7	CTRL	4_Spo_CAT	20.61329	20.65171
20	B8	CTRL	4_Spo_CAT	20.68869	20.65171
21	B9	CTRL	4_Spo_CAT	20.65316	20.65171
79	G7	CTRL	5_Spo_GST	21.10453	21.31822
80	G8	CTRL	5_Spo_GST	21.58456	21.31822
81	G9	CTRL	5_Spo_GST	21.26559	21.31822

Slice	Count	Total Area	Average Size	%Area	Mean
CO CONTROL 1 GF	8	10	1.25	0.007	238.208
CO CONTROL 1 GF	8	12	1.5	0.016	239.35
CO CONTROL 1 GF	15	25	1.667	0.032	238.754
CO CONTROL 1 GF	8	9	1.125	0.007	239.188
CO CONTROL 1 GF	1	1	1		238
CO CONTROL 1 GF	40	59	1.475	0.01	238.832
CO MD2 GREEN.tif	60	685	11.417		234.111
CO MD2 GREEN.tif	32	422	13.188		232.374
CO MD2 GREEN.tif	30	225	7.5	0.733	233.837
CO MD2 GREEN.tif	30	83	2.767	0.302	237.214
CO MD2 GREEN.tif	157	1500	9.554	0.74	234.353
CO MD2 GREEN.tif	179	1611	9	0.277	234.196
CO MD1 GREEN.tif	51	269	5.275	0.488	235.663
CO MD1 GREEN.tif	32	222	6.938	0.269	232.712
CO MD1 GREEN.tif	101	537	5.317		235.017
CO HW3 GREEN.tif	99	8382	84.667		235.605
CO HW3 GREEN.tif	211	8155	38.649	17.287	235.536
CO HW3 GREEN.tif	280	8076	28.843	16.463	235.825
CO HW3 GREEN.tif	336	11440	34.048	18.384	236.323
CO HW3 GREEN.tif	267	12435	46.573	19.363	236.231
CO HW3 GREEN.tif	360	3968	11.022		236.518
CO HW3 GREEN.tif	1250	35822	28.658		236.348
CO HW Lanthanum	332	11558	34.813	24.317	236.759
CO HW Lanthanum	307	7401	24.107	20.715	237.141
CO HW Lanthanum	285	4642	16.288		236.801
CO HW Lanthanum	395	5607	14.195		236.961
CO HW Lanthanum	425	6113	14.384		236.953
CO HW Lanthanum	350	5704	16.297		236.709
CO HW Lanthanum	435	7056	16.221		236.901
CO HW Lanthanum	679	12145	17.887	16.943	236.954
CO HW Lanthanum	455	5508	12.105		236.856
CO HW Lanthanum	2336	42377	18.141		236.9
CO HW Lanthanum	521	15646	30.031	28.231	232.574
CO HW Lanthanum	313	9379	29.965	20.671	231.812
CO HW Lanthanum	325	13273	40.84	17.616	231.915
CO HW Lanthanum	301	11350	37.708	22.624	232.462
CO HW Lanthanum	191	5559	29.105	14.381	233.114
CO HW Lanthanum	229	5382	23.502		232.94
CO HW Lanthanum	115	2207	19.191		232.492
CO HW Lanthanum	1422	43558	30.632		232.614
